# Effects of Whey Protein Supplementation Pre- or Post-Resistance Training on Muscle Mass, Muscular Strength, and Functional Capacity in Pre-Conditioned Older Women: A Randomized Clinical Trial

**DOI:** 10.3390/nu10050563

**Published:** 2018-05-03

**Authors:** Hellen C. G. Nabuco, Crisieli M. Tomeleri, Paulo Sugihara Junior, Rodrigo R. Fernandes, Edilaine F. Cavalcante, Melissa Antunes, Alex S. Ribeiro, Denilson C. Teixeira, Analiza M. Silva, Luís B. Sardinha, Edilson S. Cyrino

**Affiliations:** 1 Federal Institute of Science and Technology of Mato Grosso, Highway BR-364, Km 329, Cuiabá, Mato Grosso 78106-970, Brazil; 2 Metabolism, Nutrition, and Exercise Laboratory, Physical Education and Sport Center, Londrina State University, Highway Celso Garcia Cid, Londrina, Paraná 86057-970, Brazil; crisielitomeleri@gmail.com (C.M.T.); juniornutricao@hotmail.com (P.S.J.); rodrigo.r.fernandes@gmail.com (R.R.F); edilainefungari@gmail.com (E.F.C.); melissa.antunes@hotmail.com (M.A.); denict@uel.br (D.C.T.); edilsoncyrino@gmail.com (E.S.C.); 3 Faculty of Physical Education, University of Campinas, Érico Veríssimo avenue, Campinas, São Paulo 13083-970, Brazil; 4 Center for Research in Health Sciences, University of Northern Paraná, Paris Avenue, Londrina, Paraná 86041-140, Brazil; alex-silvaribeiro@hotmail.com; 5 Department of Physical Education, Faculty of Physical Education and Sport, State University of Londrina, Londrina, Highway Celso Garcia Cid, Londrina, Paraná 86057-970, Brazil; 6 Exercise and Health Laboratory, CIPER, Faculdade de Motricidade Humana, Universidade de Lisboa, Estrada da Costa, Cruz Quebrada, Dafundo 1499-002, Portugal; analiza@fmh.ulisboa.pt (A.M.S.); lbsardinha55@gmail.com (L.B.S.)

**Keywords:** aging, strength training, hypertrophy, protein timing

## Abstract

Aging is associated with sarcopenia and dynapenia, with both processes contributing to functional dependence and mortality in older adults. Resistance training (RT) and increased protein intake are strategies that may contribute to health improvements in older adults. Therefore, the aim was to investigate the effects of whey protein (WP) supplementation consumed either immediately pre- or post-RT on skeletal muscle mass (SMM), muscular strength, and functional capacity in pre-conditioned older women. Seventy older women participated in this investigation and were randomly assigned to one of three groups: whey protein pre-RT and placebo post-RT (WP-PLA, *n* = 24), placebo pre-RT and whey protein post-RT (PLA-WP, *n* = 23), and placebo pre- and post-RT (PLA-PLA, *n* = 23). Each group ingested 35 g of WP or PLA. The RT program was carried out over 12 weeks (three times per week; 3 × 8–12 repetition maximum). Body composition, muscular strength, functional capacity, and dietary intake were assessed pre- and post-intervention. Two-way analysis of covariance (ANCOVA) for repeated measures, with baseline scores as covariates were used for data analysis. A time vs. group interaction (*p* < 0.05) was observed with WP-PLA and PLA-WP presenting greater increases compared with PLA-PLA for SMM (WP-PLA = 3.4%; PLA-WP = 4.2%; PLA-PLA = 2.0%), strength (WP-PLA = 8.1%; PLA-WP = 8.3%; PLA-PLA = 7.0%), and the 10-m walk test (WP-PLA = −10.8%; PLA-WP = −11.8%; PLA-PLA = −4.3%). Whey protein supplementation was effective in promoting increases in SMM, muscular strength, and functional capacity in pre-conditioned older women, regardless of supplementation timing. This trial was registered at ClinicalTrials.gov: NCT03247192.

## 1. Introduction

Functional dependence, the risk of falls, and development of diseases and mortality occur in older adults during the aging process due to declines in muscle mass (sarcopenia) and muscular strength (dynapenia) [1,2]. Resistance training (RT) has been widely recommended as an effective strategy to attenuate the deleterious effects of aging [3,4]. Nutritional interventions, using protein supplementation, have been shown to counteract the effects of sarcopenia and dynapenia in older adults [5,6,7,8] and, hence, might decrease the risk of functional limitations [9].

Whey protein (WP) intake plays an important role by providing easy digestion and a higher peak of circulating amino acids, with leucine as the key amino acid for activation of muscle protein synthesis [5]. It can be assumed that a greater amount of leucine could result in a higher rate of protein synthesis when associated with a training stimulus, which may attenuate the losses related to aging. Information regarding protein supplementation in older adults is still controversial, mainly on the effectiveness of protein supplementation on muscle mass and strength when associated with training [6,10,11,12]. Possible explanations for these conflicting results are the anabolic stimulus from the food and training together, the amount of baseline protein consumption, the level of training, and the timing of nutrient intake [12,13,14,15,16].

Regarding the timing of nutrient intake, the hypothesis of the time of protein intake is based on the window of opportunity, with the objective of maximizing RT-induced adaptations and optimizing recovery tissue damage [16]. An increase in the availability of amino acids in the bloodstream may attenuate the catabolic effect from RT, thus contributing to an increase in skeletal muscle mass (SMM) and muscular strength, and also improving functional capacity, since adequate protein intake has been associated with the aforementioned conditions [17,18].

Only three longitudinal studies have investigated the effects of timing of protein intake on untrained older people, and with conflicting results. Candow et al. [19] showed no significant differences in SMM or muscular strength between protein supplementation before or after training. Verdijk et al. [20] found that protein supplementation immediately before and after RT did not enhance SMM or muscular strength. On the other hand, Esmarck et al. [21] investigated delayed protein intake, and demonstrated that over 12 weeks of RT, SMM increased when protein was ingested immediately after exercise, whereas no significant changes were observed when protein was supplemented 2 h post-exercise. Despite these findings, such studies were conducted with untrained older adults, and conservative doses of administrated protein, which is not ideal for older people (10 to 20 g). Thus, it remains unclear whether training status influences the hypertrophic response to optimal dose (30 to 40 g) pre- or post-exercise, since muscle adaptations tend to be larger in novices [16].In young adults, Cribb and Hayes (2006) [22] showed that the protein supplement timing represents a simple but effective strategy to enhance the adaptations (strength and hypertrophy) that are desired from RT. On the other hand, Hoffman et al. (2009) [23] and Schoenfeld et al. (2017) [24] showed no significant differences in timing protein intake muscular adaptations in well-trained men. Thus, training status had not yet been tested in women and/or in the elderly.

Our laboratory recently showed, in another cohort of women [8], that 12 weeks of WP supplementation post-RT performed three times per week improved SMM and muscular strength in pre-conditioned older women. However, in this previous study, we were unable to analyze the impact of timing of protein intake. Therefore, the aims of the present study were: (1) to determine whether our previous findings that WP supplementation post-RT improved SMM and muscular strength would be replicated in a larger cohort of pre-conditioned older women; and (2) to investigate the effects of WP supplementation consumed either immediately pre- or post-RT on SMM, muscular strength, and functional capacity in pre-conditioned older women. Based on the previous findings, we hypothesized that, independent of the timing of consumption, WP supplementation would result in greater gains in SMM and muscular strength with a consequent improvement in functionality than the placebo, in a sample of pre-conditioned elderly women.

## 2. Material and Methods

### 2.1. Experimental Design

This three-arm randomized, double-blind, placebo-controlled design was carried out over a period of 26 weeks, divided into two phases. The first phase of the study was an eight-week period (weeks 3–10), during which participants were familiarized with RT. This RT period was conducted to standardize the training status, and to overcome strong neural adaptations known to occur within the first few weeks of RT [25].

**Figure 1:**
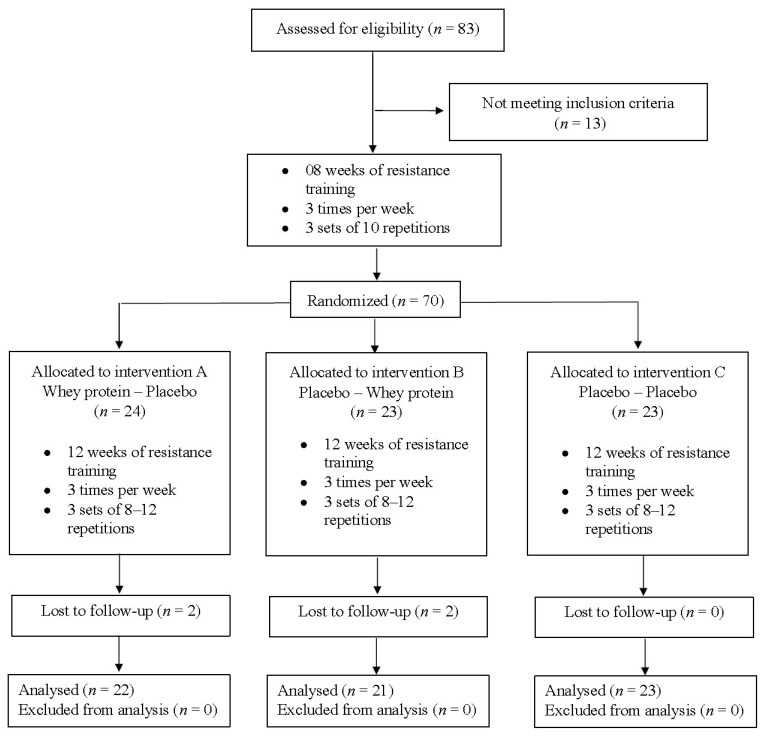
Flow chart of the study.

In Phase 2, the supplementation phase, the participants were randomized into three groups and started 12 weeks dedicated to WP supplementation plus RT (weeks 13–24). At the beginning and end of each phase of the experiment, two weeks were allocated for evaluations consisting of anthropometric (weeks 2, 12, and 26), body composition (weeks 2, 12 and 26), one repetition maximum tests (weeks 1, 11, and 25), and dietary intake measurements (weeks 1, 11, and 25). The anthropometric, body composition, and dietary intake measurements were carried out in a temperature-controlled room (22–24 °C), and the RT sessions were conducted at the university training facility. Figure 1 presents a schematic representation of the women recruitment and allocation adopted in this investigation.

### 2.2. Participants

The present study is part of the Active Aging Project, a longitudinal cohort study designed to examine the role of RT on older women’s health. Recruitment was carried out through newspaper and radio advertising, and home delivery of leaflets in the central area and residential neighborhoods. All participants completed health history and physical activity questionnaires and met the following inclusion criteria: 60 years old or more, physically independent, free from cardiac or orthopedic dysfunction, not receiving hormonal replacement and/or thyroid therapy, not using equipment that would prevent the accomplishment of protocols and tests, and not having performed any regular physical exercise for six months preceding the beginning of the study. Participants passed a diagnostic graded exercise stress test with 12-lead electrocardiogram reviewed by a cardiologist and were released with no restrictions for participation in this investigation.

Eighty-three Brazilian older women (≥60 years old) volunteered to participate in this investigation. After individual interviews, thirteen volunteers were excluded as they did not meet the inclusion criteria. Seventy participants were submitted to a standardized resistance training (RT) program, for eight weeks. After the assessments, the participants were randomly divided into three groups according to relative strength (ratio of total strength obtained in 1-repetition maximum test by body mass): (1) whey protein pre- and placebo post-RT (WP-PLA, *n* = 24, 67.5 ± 5.2 years, 69.0 ± 14.8 kg, 26.4 ± 5.2 kg/m^2^); (2) placebo pre- and whey protein post-RT (PLA-WP, *n* = 23, 66.2 ± 9.4 years, 65.4 ± 16.7 kg, 25.3 ± 5.4 kg/m^2^); and (3) placebo pre- and post-RT (PLA-PLA, *n* = 23, 66.5 ± 7.2 years, 62.2 ± 10.4 kg, 23.8 ± 3.7 kg/m^2^). A blinded researcher was responsible for generating random numbers for participant allocation. All groups were submitted to the same RT program and 66 participants completed the experiment. The reasons for withdrawal from the study were reported as personal reasons and transportation issues.

Written informed consent was obtained from all participants after a detailed description of investigation procedures had been provided. This investigation was conducted according to the Declaration of Helsinki and approved by the local University Ethics Committee (No. 1.700.756).

### 2.3. Anthropometry

Body mass was measured to the nearest 0.1 kg using a calibrated electronic scale (Balmak, Laboratory Equipment Labstore, Curitiba, PR, Brazil), with participants wearing light workout clothing and no shoes. Height was measured using a stadiometer to the nearest 0.1 cm while subjects were standing without shoes.

### 2.4. Body Composition

Whole-body dual-energy X-ray absorptiometry (DXA) (Lunar Prodigy, model NRL 41990, GE Lunar, Madison, WI, USA) was used to assess lean mass, both total and segmented, according to previously described procedures [26]. SMM was estimated by the predictive equation proposed by Kim et al. [27]. Previous test-retest scans of 12 older women measured 24–48 h apart resulted in a standard error of measurement (SEM) of 0.24 kg for SMM, 0.20 for lower limb lean soft tissue (ULLST), and 0.10 kg for upper limb lean soft tissue (ULLST), with an intraclass correlation coefficient (ICC) > 0.99 for all variables.

### 2.5. Muscular Strength

Maximal dynamic strength was evaluated using the 1 RM tests assessed in the chest press (CP), knee extension (KE), and preacher curl (PC) exercises, performed in this order, according to previously-described procedures [8]. The 1 RM was recorded as the final load lifted in which the participant was able to complete only one single maximal execution. Three 1-RM sessions were performed separated by 48 h. The highest load achieved in the three sessions was used for the analysis in each exercise. Total strength was determined by the sum of the three exercises and divided by body mass to estimate the relative total strength. The SEMs for the CP, KE, and PC were 0.46 kg, 1.67 kg, and 0.93 kg, respectively, and the ICCs for the CP, KE, and PC were 0.97, 0.91, and 0.93, respectively.

### 2.6. Functional Capacity

Functional capacity was evaluated using two tests from the Latin American Development Group for Maturity protocol [28]: the 10-m walk test (10 MW) and rising from the sitting position test (RSP). For the 10 MW, participants were required to walk a distance of 10 m rapidly to evaluate gait speed; and for the RSP, they were required to stand up and sit down five times as quickly as possible. The rest interval between tests was 3 min. A digital timer was used to record the time of the tests. The SEMs for the 10 MW and RSP were 0.12 s and 0.15 s, with ICCs of 0.94 and 0.98, respectively.

### 2.7. Dietary Intake

Food consumption was assessed by the 24-h dietary recall method, applied on two non-consecutive days of the week. A photographic manual of food portion sizes was used to improve the precision of dietary intake reporting [29]. The homemade measurements of nutritional values of food and supplementation were converted into grams and milliliters using the online software Virtual Nutri Plus (Keeple^®^, Rio de Janeiro, Brazil) for diet analysis. Some foods were not found in the program database and were added from food tables [30].

### 2.8. Supplementation Protocol

Participants received a dose of 35 g of hydrolyzed WP (Lacprodan^®^, Arla Foods, Sønderhøj, Denmark) and/or placebo pre and post-RT. Maltodextrin (New Millen^®^, São Paulo, SP, Brazil) was used as a placebo. The hydrolyzed WP drink contained 27.1 g of protein, 5.2 g of carbohydrates, and 0.2 g of fat per portion (200 mL, 131 kcal), whereas the carbohydrate drink contained 0.3 g of protein and 33.3 g of carbohydrates per portion (200 mL, 134 kcal). The supplements were mixed with non-caloric sugar-free drinks to mask the contents (grape or passion fruit flavor). Participants ingested the drinks under the supervision of the study staff and were instructed to drink the solution as quickly as possible. Participants were instructed to avoid eating 1 h before the supplementation pre-RT and 1 h after supplementation post-RT. Supplementation was only consumed on training days. Both the subjects and the researchers responsible for the RT were blinded as to which supplement was given until the end of the trial.

### 2.9. Resistance Training Program

Supervised RT was performed during the morning hours. The protocol was based on recommendations for RT in an older population to improve muscular strength and hypertrophy [3,4]. Physical education professionals with substantial RT experience personally supervised all participants to help ensure consistent and safe exercise performance. In the two phases, the sessions were performed three times per week on Mondays, Wednesdays, and Fridays. The RT program was a whole-body program with eight exercises, including: the chest press, horizontal leg press, seated row, knee extension, preacher curl (free weights), leg curl, triceps pushdown, and seated calf raise. During the pre-supplementation training period (first phase), the participants performed a conventional RT, alternated by segment, which consisted of the execution of three series of 10 maximal repetitions. During the supplementation plus RT period, the participants were submitted to a conventional RT, alternated by segment, which consisted of the execution of three series of 8 to 12 RM, with fixed loads. Throughout the investigation, instructors adjusted the loads of each exercise according to the subject’s abilities and improvement in exercise capacity in order to ensure that the subjects exercised with as much resistance as possible while maintaining proper exercise technique. The load was adjusted weekly using procedures described elsewhere [3]. During each RT session, researchers wrote down the load performed by participants for each exercise. Thus, the sum of the load used in the three sets of the eight exercises was considered the total session load, and then the weekly training load was determined by the sum of the three sessions of a week [31].

## 3. Statistical Analyses

The Shapiro–Wilk test was used to test data distribution. Data are presented as means, standard deviations, and percentage changes. The paired *t*-test was used to analyze the effects (pre- vs. post-) of the first phase. For the second phase, two-way analysis of covariance (ANCOVA) for repeated measures was applied for comparisons, with baseline scores used as covariates. When the F-ratio was significant, Bonferroni’s post hoc test was employed to identify the mean differences. The effect size (ES) was calculated to verify the magnitude of the differences by Cohen’s d, where an ES of 0.20–0.49 was considered as small, 0.50–0.79 as moderate, and ≥0.80 as large [32]. For all statistical analyses, significance was accepted at *p* < 0.05. The data were analyzed using SPSS software version 20.0 (SPSS, Inc., Chicago, IL, USA).

The sample size estimation was conducted using G*Power (version 3.0.10, Universitat Kiel, Kiel, Germany). Data from a previous investigation [33] was utilized to perform the sample size estimation. We based the calculation on an effect size of 0.33, α level of 0.05, and power (1 − β) of 80%, giving a total of 72 volunteers required. Considering a drop-out of ~15%, we recruited 83 older women.

## 4. Results

The first eight weeks of the RT period were used to standardize the training level of the participants. There was a significant (*p* < 0.05) increase in SMM, ULLST, lower limb lean soft tissue (LLLST), and muscular strength, and an improvement in functional tests. There were no significant (*p* > 0.05) main effects in habitual intake during the first phase (Table 1).

Daily intake of total energy and macronutrients at pre-, during, and post- 12 week RT are shown in Table 2. There were no significant (*p* > 0.05) main effects, indicating that the habitual daily energy and macronutrients intake were not different between groups and did not change over time. When dietary intake from food was combined with supplement intake total energy intake was increased at 12 weeks in all groups (*p* < 0.05) and was not different between groups (*p* > 0.05). As expected, significant differences among groups were reveled (*p* < 0.05) for both carbohydrate and protein intake at the post-training condition, in which the WP groups ingested higher amounts of protein, while the PLA-PLA ingested higher amounts of carbohydrate (Table 2).

Body composition, muscular strength, and functional capacity outcomes (12-weeks) are presented in Table 3. A significant (*p* < 0.001) time vs. group interaction was observed for SMM, LLLST, CP, KE, and total strength (TS), with both the WP-PLA and PLA-WP presenting greater increases compared with the PLA-PLA, without differences between the timing of protein intake. A significant (*p* < 0.05) time vs. group interaction was observed in the 10 MW, with both WP groups presenting significantly decreased scores compared to the PLA-PLA. In all groups, a main effect for time (*p* < 0.05) was observed for ULLST, PC, RSP, and training load with similar improvements.

**Figure 2:**
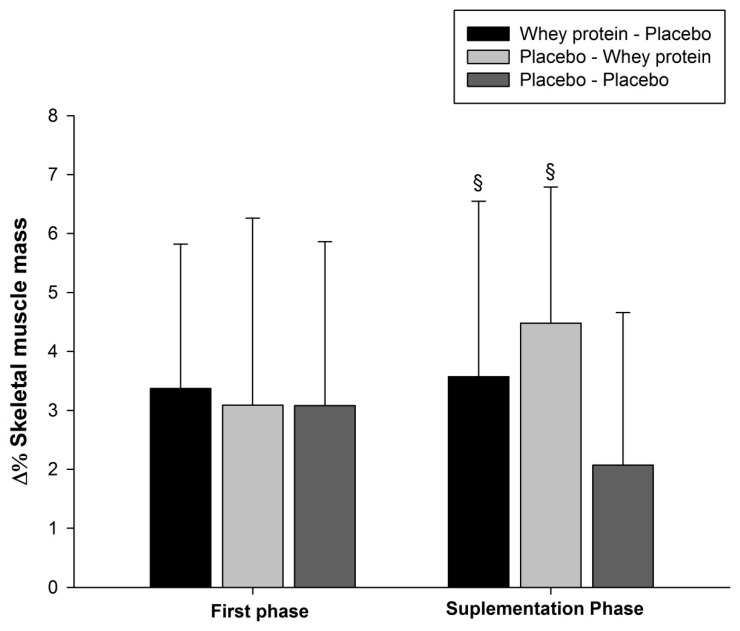
Relative skeletal muscle mass changes from the first phase and the supplementation phase. During the first phase no supplementation was provided, data are presented separately to show that both groups demonstrated the same behavior during the pre-supplementation phase in the same training phase. ^§^ *p* < 0.05 vs. placebo-placebo.

During the first phase, the changes in SMM were similar between groups (*p* > 0.05), however, after supplementation phase, a significant increase was found (*p* ≤ 0.05) in the WP-PLA and PLA-WP groups compared to the PLA-PLA (Figure 2).

## 5. Discussion

This is the first randomized double-blind controlled trial to investigate the effects of WP intake pre- or post-RT on pre-conditioned older women. The major finding of the current investigation was that WP supplementation increased SMM, ULLST, and functional capacity in pre-conditioned older women, independent of the time of administration around each training session. Our hypothesis was confirmed, as SMM, muscular strength, and functional capacity improved in the protein supplementation groups when compared to the PLA-PLA. Our study corroborates with previous results from our laboratory that showed that WP post-RT improved SMM and muscular strength in pre-conditioned older women [8].

In this study, we were able to stabilize the initial gains provided by RT, as participants underwent an initial eight weeks of RT. At the beginning of RT, the preliminary adjustment will be neurological. The initial increase in muscular strength occurs more rapidly than muscle hypertrophy, relating to motor learning [34,35]. After these neural adaptations, in a progressive way, the muscular hypertrophy starts to exert a larger part of the contribution in the increases of muscular strength [36]. Supplementation was provided only after this initial phase. The design of this study enabled us to isolate the effects of supplementation. Naclerio and Larumbe-Zabala [11] conducted a meta-analysis on the effects of WP supplementation on trained individuals. The authors reported that WP supplementation in resistance-trained individuals favors superior gains in lean body mass and muscular strength, in young and middle-age adults, however, the numbers of studies with trained individuals are scarce, especially among older adults. Morton et al. have shown that training experience increases the effectiveness of protein supplementation in body composition and muscular strength, and that the increase in fat-free mass is larger in trained individuals [12]. Bell et al. [37] showed that WP plus creatine was effective for increasing lean body mass and total muscular strength in older men, however, when starting an exercise program, protein supplementation was no longer relevant.

If adequate protein and energy intake occurs in untrained individuals starting a RT program, the stimulation provided by RT overcomes the stimulus coming from diet [15]. Therefore, the lack of favorable results for WP supplementation associated with RT [10] may be due to the competition of anabolic stimuli (feeding and training together).

On training days, our participants reached an average protein intake of 1.5 g/body weight/day (habitual intake + supplementation). This amount is in accordance with the protein recommendations that have been proposed for the elderly [38]. Previous meta-analysis reported that a protein dose above 1.2 g/body weight/day is sufficient to increase fat-free mass in older people [6]. Moreover, higher quality protein was provided, as WP, especially hydrolyzed, promotes rapid release of amino acids into the bloodstream [5], a very important feature for improving the digestion process [39]. WP contains high quantities of branched chain amino acids, in particular leucine, a key amino acid for the activation of muscle protein synthesis [5]. It has been suggested that 30 to 35 g of WP is required to provide appropriate stimulation of postprandial muscle protein synthesis in older individuals [40,41]. We provided a 35 g dose of WP, containing 3.8 g of leucine, an amount expected to be sufficient to promote protein synthesis stimulation (≥2 g leucine) [12,42].

We showed that protein supplementation provides a larger increase in muscular strength when compared with a placebo, providing a possible explanation for the better performance in the functional tests. These results are in agreement with previous studies with untrained individuals [43,44] and trained [8] elderly people. Indeed, gains in strength are extremely important for the health status in older adults, as strength improvements are associated with a reduced risk of mortality [45], as well as higher lean body mass [46].

We observed improvements in functional capacity, specifically for the 10 WM-test, across the groups, with large increases in the protein groups when compared with the placebo. Improvements in functional capacity are of important clinical relevance and reduce the risk of institutionalization and mortality [47]. It could be speculated that such improvements in functional capacity in the WP-PLA and PLA-WP are due to improvements in SMM (which influences muscle quality) and neuromuscular action [9,48], and also due to improvements in muscular strength [49]. In fact, Liao et al. have shown protein supplementation combined with RT promoted improvements in lean soft tissue, muscular strength, and functional capacity [44]. However, the effect on strength gain was not associated with improvements in most components of physical mobility [44]. On the other hand, the authors state that there are few available studies comparing protein supplementation and RT with physical function and mobility. With respect to protein timing, although the SMM, functional capacity, and total strength gains were higher in the PLA-WP, no significant differences were observed between the WP groups. These results are in agreement with previous studies with older adults [19,20] and well-trained young individuals [24]. Moreover, Dideriksen et al. have shown protein supplementation 30 min before or after exercise did induce a solid anabolic stimulus, which persisted beyond the time point where the essential amino acids’ availability had returned to basal concentrations [50]. We used maltodextrin to blind the study and to promote an isocaloric condition. Maltodextrin has been used as a control in several studies, since it is a good source of energy. However, despite carbohydrate supplementation stimulating a greater insulin release than protein supplementation, the insulin is not as effective as amino acids in stimulating muscle anabolic pathways [51], therefore, maltodextrin does not play an important role in muscle hypertrophy. On the other hand, carbohydrate supplementation can improve physical performance, but in exercise of less than 1 h, muscle glycogen is not limiting and the type and (or) amount of carbohydrates (CHO) appears to be irrelevant to improve physical performance [52,53].

This study is not without limitations. The results are specific to healthy pre-conditioned older women and should not be extrapolated to other populations. Physical activity was not assessed during the free living conditions, although additional RT, beyond that provided by the university, was not expected. Nevertheless, the present study is the first to investigate the effects of WP supplementation pre- and post-RT on SMM, muscular strength, and functional capacity in pre-conditioned older women. We also monitored dietary intake pre-, during, and post-intervention ensuring the participants did not change their habitual intake throughout the study, even after the supply of supplements.

In conclusion, this investigation showed that WP supplementation pre- or post-RT is effective in promoting increases in SMM, LLLST, muscular strength, and functional capacity in pre-conditioned older women. Thus, in clinical practice, WP intake, before or after RT, is a strategy that may be adopted to prevent sarcopenia and dynapenia, and also improve physical functioning of older adult women. Further studies are necessary to investigate the timing of protein intake based on pre-sleep supplementation protein, since resistance training during the day increases the overnight muscle protein synthetic response [54].

## Figures and Tables

**Table 1:** Participant scores at baseline (pre) and after (post) the eight-week period (*n* = 70).

	Pre	Post	Δ%	ES	*p*
Protein (g/kg/day)	0.92 ± 0.28	0.94 ± 0.28	2.2	0.07	0.499
CHO (g/kg/day)	3.07 ± 1.0	3.15 ± 1.0	2.5	0.08	0.324
Lipids (g/kg/day)	0.70 ± 0.2	0.73 ± 0.2	4.5	0.14	0.233
Energy (kcal/kg/day)	22.3 ± 6.5	23.0 ± 6.5	3.1	0.10	0.178
SMM (kg)	16.6 ± 2.6	17.1 ± 2.7	3.1	0.19	<0.001
ULLST (kg)	3.8 ± 0.6	4.0 ± 0.6	3.5	0.21	<0.001
LLLST (kg)	11.5 ± 1.6	11.8 ± 1.7	2.5	0.17	<0.001
Chest press (kg)	40.6 ± 8.5	44.8 ± 8.7	10.3	0.48	<0.001
Knee extension (kg)	46.0 ± 11.3	52.6 ± 11.7	14.4	0.57	<0.001
Preacher curl (kg)	18.4 ± 3.8	21.8 ± 3.7	18.3	0.89	<0.001
Total strength (kg)	105.0 ± 19.7	119.3 ± 20.3	13.6	0.71	<0.001
10 MW (s)	7.8 ± 1.1	7.4 ± 0.9	−4.8	0.37	<0.001
RSP (s)	12.4 ± 2.2	11.8 ± 1.7	−5.2	0.33	<0.001

Paired *t*-test. Data are expressed as mean and standard deviation. CHO = carbohydrates; ES = effect size; SMM = skeletal muscle mass; ULLST = upper limb lean soft tissue; LLLST = lower limb lean soft tissue; 10 MW = 10-m walk test; RSP = rising from sitting position.

**Table 2:** Habitual dietary intake of the older women measured at baseline, during, and after 12 weeks of intervention (*n* = 66).

	Whey Protein–Placebo (*n* = 22)			Placebo–Whey Protein (*n* = 21)			Placebo-Placebo (*n* = 23)			Interaction *p*-Value
	0	6-Week	12-Week	0	6-Week	12-Week	0	6-Week	12-Week	
Intake excluding whey/control										
Protein (g/kg/day)	0.92 ± 0.20	0.95 ± 0.36	0.96 ± 0.19	0.94 ± 0.36	0.98 ± 0.52	0.98 ± 0.24	0.95 ± 0.27	0.96 ± 0.26	0.99 ± 0.25	0.914
CHO (g/kg/day)	3.1 ± 0.96	3.1 ± 0.91	3.0 ± 0.77	3.2 ± 1.12	3.1 ± 1.24	3.1 ± 1.13	3.1 ± 0.94	3.1 ± 0.84	3.1 ± 0.72	0.968
Lipids (g/kg/day)	0.77 ± 0.29	0.72 ± 0.27	0.69 ± 0.19	0.76 ± 0.30	0.72 ± 0.21	0.74 ± 0.32	0.67 ± 0.17	0.73 ± 0.14	0.75 ± 0.34	0.485
Energy (kcal/kg/day)	22.9 ± 6.29	23.1 ± 7.76	23.2 ± 6.28	22.4 ± 7.75	23.3 ± 7.76	23.3 ± 6.28	22.4 ± 5.90	23.2 ± 4.6	23.4 ± 6.3	0.810
Intake including whey/control										
Protein (g/kg/day)	0.92 ± 0.20	1.38 ± 0.42 *^,§^	1.38 ± 0.26 *^,§^	0.94 ± 0.36	1.42 ± 0.57 *^,§^	1.49 ± 0.46 *^,§^	0.95 ± 0.27	0.98 ± 0.30	1.0 ± 0.25	<0.001
CHO (g/kg/day)	3.1 ± 0.96	3.7 ± 0.93 *	3.6 ± 0.85 *^,§^	3.2 ± 1.12	3.4 ± 1.5 ^§^	3.6 ± 1.2 *^,§^	3.1 ± 0.94	4.2 ± 1.0 *	4.2 ± 0.90 *	<0.001
Lipids (g/kg/day)	0.77 ± 0.29	0.72 ± 0.27	0.70 ± 0.17	0.76 ± 0.30	0.70 ± 0.29	0.74 ± 0.31	0.67 ± 0.17	0.72 ± 0.14	0.75 ± 0.33	0.279
Energy (kcal/kg/day)	22.9 ± 6.29	26.6 ± 7.0	26.1 ± 5.6 *	22.4 ± 7.75	25.3 ± 10.4	28.0 ± 6.7 *	22.4 ± 5.90	27.2 ± 4.6 *	27.8 ± 6.8 *	0.435

Analysis of variance (ANOVA) two-way. Data are expressed as mean and standard deviation. ES = effect size; CHO = carbohydrates. * *p* < 0.05 vs. pre training; ^§^ *p* < 0.05 vs. placebo-placebo.

**Table 3:** Body composition and muscular strength of the older women after the 12-week intervention period (*n* = 66).

	Whey Protein–Placebo (*n* = 22)				Placebo–Whey Protein (*n* = 21)				Placebo-Placebo (*n* = 23)				Interaction *p*-Value
	Pre	Post	Δ%	ES	Pre	Post	Δ%	ES	Pre	Post	Δ%	ES	
SMM (kg)	17.7 ± 2.5	18.4 ± 2.4 *^,^^§^	3.4 ± 2.9	0.25	17.4 ± 3.2	18.2 ± 3.2 *^,^^§^	4.2 ± 2.3	0.23	16.2 ± 2.2	16.6 ± 2.2 *	2.0 ± 2.1	0.14	<0.001
ULLST (kg)	4.1 ± 0.47	4.2 ± 0.45 *	3.4 ± 3.0	0.29	3.9 ± 0.54	4.1 ± 0.58 *	5.9 ± 4.3	0.40	3.7 ± 0.53	3.9 ± 0.53 *	4.1 ± 3.5	0.29	0.156
LLLST (kg)	11.8 ± 1.2	12.1 ± 1.1 *^,^^§^	3.2 ± 2.9	0.30	11.6 ± 1.4	12.0 ± 1.5 *^,^^§^	3.7 ± 2.2	0.30	11.3 ±1.4	11.4 ± 1.4 *	1.1 ± 2.2	0.08	<0.001
CP (kg)	46.0 ± 9.0	49.0 ± 10.0 *^,^^§^	5.6 ± 1.7	0.28	45.0 ± 9.0	48.0 ± 10.0 *^,^^§^	5.9 ± 1.6	0.28	43.0 ± 8.0	45.0 ± 8.0 *	4.5 ± 1.2	0.24	<0.05
KE (kg)	52.0 ± 11.0	56.0 ± 12.0 *^,^^§^	9.2 ± 2.5	0.39	55.0 ± 11.0	59.0 ± 12.0 *^,^^§^	8.8 ± 2.2	0.41	52.0 ± 13.0	56.0 ± 13.0 *	7.5 ± 1.0	0.32	<0.001
PC (kg)	23.0 ± 4.0	25.0 ± 4.0 *	11.3 ± 5.7	0.59	22.0 ± 3.0	25.0 ± 4.0 *	12.4 ± 6.6	0.75	21.0 ± 3.0	23.0 ± 4.0 *	10.5 ± 5.3	0.63	0.376
TS (kg)	121.0 ± 20.0	131.0 ± 21.0 *^,^^§^	8.1 ± 1.6	0.48	122.0 ± 21.0	132.0 ± 22.0 *^,^^§^	8.3 ± 2.3	0.47	115.0 ± 21.0	124.0 ± 22.0 *	7.0 ± 2.7	0.38	<0.05
Training load (kg)	1735 ± 232	2505 ± 292 *	45.3 ± 14.8	2.93	1698 ± 224	2429 ± 377 *	43.6 ± 18.3	2.44	1630 ± 276	2367 ± 442 *	44.7 ± 14.2	2.05	0.916
10 MW (s)	7.5 ± 0.9	6.7 ± 0.9 *^,^^§^	−10.8 ± 11.3	0.90	7.5 ± 1.0	6.6 ± 1.0 *^,^^§^	−11.8 ± 8.6	0.89	7.3 ± 0.9	6.9 ± 0.7 *	−4.3 ± 8.4	0.41	<0.05
RSP (s)	12.0 ± 1.6	10.8 ± 1.1 *	−10.0 ± 12.4	0.89	11.7 ± 1.5	10.5 ± 1.7 *	−10.1 ± 5.4	0.73	11.7 ± 1.8	11.0 ± 1.8 *	−5.7 ± 7.6	0.36	0.176

ANCOVA two-way. Data are expressed as mean and standard deviation. ES = effect size; SMM = skeletal muscle mass; ULLST = upper limb lean soft tissue; LLLST = lower limb lean soft tissue; CP = Chest press; KE = Knee extension; PC = Preacher curl; TS = total strength; 10 MW = 10-m walk test; RSP = rising from sitting position.* *p* < 0.05 vs. pre training; ^§^ *p* < 0.05 vs. placebo-placebo.
